# The influence of hepatitis B and C virus coinfection on liver histopathology in children

**DOI:** 10.1007/s00431-014-2402-7

**Published:** 2014-08-30

**Authors:** Maria Pokorska-Śpiewak, Barbara Kowalik-Mikołajewska, Małgorzata Aniszewska, Bożena Walewska-Zielecka, Magdalena Marczyńska

**Affiliations:** 1 Department of Children’s Infectious Diseases, Medical University of Warsaw, ul. Wolska 37, 01-201 Warsaw, Poland; 2 Warsaw Hospital for Infectious Diseases, Warsaw, Poland; 3 Department of Public Health, Medical University of Warsaw, Warsaw, Poland

**Keywords:** Chronic hepatitis, Grading and staging, Hepatitis B virus, Hepatitis C virus, Liver biopsy

## Abstract

The influence of hepatitis B virus (HBV) and hepatitis C virus (HCV) coinfection on liver histology in children remains unknown. We analyzed histopathological features in 70 treatment-naïve children: 10 with HBV/HCV coinfection (case group A), 30 with HBV (control group B), and 30 with HCV (control group C). Liver biopsies were scored for grading and staging according to Knodell’s modified system and were tested for an association with demographic and laboratory data. The mean grade was higher in coinfected children compared to control group C (6.2 ± 3.0 vs. 4.2 ± 2.5, *p* = 0.04), but not control group B (*p* = 0.47). A higher proportion of patients with moderate to severe necroinflammation were observed in case group A compared to isolated HCV (*p* = 0.05). Mean staging did not differ between the case and control groups. Multivariate analysis revealed that HBV/HCV coinfection and aminotransferase activity were independently associated with moderate to severe necroinflammatory activity

*Conclusion*: HBV/HCV coinfection was associated with moderate to severe necroinflammation irrespective of age at biopsy or duration of infection and led to significantly higher necroinflammatory activity than HCV monoinfection. HBV/HCV coinfection did not enhance fibrosis. High aminotransferase levels were positively associated with moderate to severe necroinflammation.

## Introduction

Hepatitis B virus (HBV) and hepatitis C virus (HCV) are the most common causes of chronic liver disease, leading to important global public health problems [28]. According to the World Health Organization (WHO), 350 million people worldwide are chronically infected with HBV and 170 million with HCV [4, 28]. Because the two viruses share modes of transmission, HBV and HCV coinfection is rather common, especially in endemic areas and among patients with a high risk of parenteral infections [4, 17]. Approximately 2–10 % of HCV-infected individuals are HBsAg-positive, whereas the presence of anti-HCV is confirmed in 5–20 % of patients with HBV infection [4, 17, 25]. However, the exact number of patients with coinfection is unknown but is thought to be higher [25]. The literature suggests that coinfection with HBV and HCV may have considerable clinical relevance. Dual infection is associated with more severe liver disease and frequent progression to cirrhosis and hepatocellular cancer [9, 23, 24]. However, some evidence exists for reciprocal replicative suppression between both viruses [2, 9]. Information concerning many aspects of coinfection is currently incomplete and controversial [23, 25]. Moreover, only sparse and inconsistent data are available regarding chronic HBV and HCV coinfection in the pediatric population [22, 26, 27]. Several studies have reported that children with chronic viral hepatitis tend to have milder disease than adults [10, 11, 15]. However, the impact of dual infection acquired in early childhood remains unknown. Therefore, the aim of the present study was to analyze the histopathological features in children with HBV and HCV coinfection and to compare them to patients with HBV or HCV monoinfection. The analysis also included factors associated with necroinflammation and fibrosis in the liver.

## Methods

### Patients

Seventy-one consecutive pediatric patients with confirmed chronic viral hepatitis undergoing liver biopsy in our department between 2001 and 2012 were analyzed retrospectively. Liver biopsy was performed as part of the qualification procedure for antiviral therapy. Patients were qualified for liver biopsy (and treatment) on the basis of the current practical guidelines of the European Association for the Study of the Liver (EASL) and according to the national therapeutic programs. Briefly, we included children over 3 years of age with the following additional criteria: (1) for HBV-infected patients, either persistently increased aminotransferase levels and/or HBV DNA levels >2,000 IU/ml, and (2) for HCV-infected children, detectable HCV RNA regardless of the aminotransferase pattern [7, 8]. HIV or HDV-infected children were not included. In addition, other well-established causes of liver disease, such as autoimmune hepatitis, Wilson’s disease, alpha 1-antitrypsin deficiency, or nonalcoholic fatty liver disease (NAFLD), were exclusion criteria for this study. Probable modes and dates of transmission were determined based on the available medical records. The age when the infection was acquired and the duration of the disease were calculated from the beginning of risk exposure. According to these data, all children with HBV/HCV coinfection were infected simultaneously with both viruses. Chronic hepatitis was diagnosed in children with >6-month history of viral hepatitis on the basis of elevated alanine and aspartate aminotransferase (ALT and AST, respectively) activities, serological testing, and nucleic acid testing. ALT and AST serum levels were measured using commercially available laboratory tests (Vitros, Ortho Clinical Diagnostics, Johnson & Johnson). For both ALT and AST, 40 IU/l was considered the upper limit of normal (ULN). Serum markers of HBV infection (HBsAg, HBeAg, anti-HBeAg, anti-HBc, anti-HBs) were measured with commercially available ELISA kits (Vitros ECi, Ortho Clinical Diagnostics, Johnson & Johnson). Anti-HCV was determined using a third-generation ELISA test (Ortho Vitros ECi, Ortho Clinical Diagnostics, Johnson & Johnson). HBV DNA and HCV RNA were detected using real-time polymerase chain reaction (RT-PCR) (Amplico, Roche, and Cobas TaqMan, Roche). Chronic hepatitis B was diagnosed on the basis of HBsAg positivity and was confirmed by PCR positive for HBV DNA, whereas chronic hepatitis C was diagnosed in anti-HCV-positive children and was confirmed by PCR positive for HCV RNA. HBV/HCV coinfection was diagnosed in children positive for both HBsAg and anti-HCV and was confirmed by the detection of HBV DNA and HCV RNA. Body mass index (BMI) standard deviation (SD) scores (BMI *z*-scores) were calculated according to the WHO Child Growth Standards and Growth reference data using the WHO anthropometric calculator AnthroPlus v.1.0.4. Obesity was diagnosed when the BMI *z*-score was >2 SD.

### Liver biopsy

Percutaneous liver biopsy was performed using the Menghini needle (Hepafix kit 1.4 or 1.6 mm, Braun). All biopsy specimens were fixed in 4 % buffered formalin and routinely processed in paraffin. Serial 4-μm-thick slides were stained with hematoxylin and eosin, impregnated with silver according to the Gomori method for reticulin fibers, and stained with Chromotrope 2R and aniline blue for collagen fibers. The histological evaluation was performed by one pathologist experienced in hepatopathology (BWZ) who was blinded to the clinical data. The histology was assessed according to Knodell’s numerical scoring system modified by international experts [3, 5, 16]. Necroinflammatory activity was graded as the final sum of points in three categories: periportal and bridging necrosis, intralobular necrosis, and portal inflammation, which gave a histologic activity index (HAI) ranging from 0 to 18 points. The necroinflammation grade was considered minimal when HAI was 0–3 points, mild if 4–8 points, moderate if 9–12 points, and severe if 13–18 points. The fibrosis stage was assessed using a five-point scale: 0, no fibrosis; 1, portal fibrosis, fibrous portal expansion; 2, periportal fibrosis, periportal or scarce portal-portal septa; 3, septal fibrosis, fibrous septa with architectural distortion; and 4, cirrhosis. In addition, we analyzed the presence of the following abnormalities: portal lymphoid aggregates/follicles (lymphadenoplasia) and steatosis.

### Statistical analysis

Data were tested for normal distribution using the Kolmogorov-Smirnov test. Continuous variables were presented as means ± SDs or medians with interquartile ranges (IQRs) and were compared using either Student’s *t* test or Mann-Whitney test. Categorical variables were compared using either the chi-square test or Fisher’s exact test, as appropriate. Even though grading and staging are both categorical variables, mean grading and staging scores were calculated as in other papers in order to make a comparison with the results of the other authors possible [21, 24, 28].

The Kruskal-Wallis test or analysis of variance was used to compare age at biopsy, age at infection acquisition, duration of infection, and ALT and AST activity in patient subgroups according to the grade and staging. Stepwise logistic regression with the accompanying *C* statistic was used to determine factors associated with moderate to severe necroinflammation (grade ≥9 points) or fibrosis (staging ≥3 points). The following variables were entered into the model: HBV/HCV coinfection, HBV monoinfection, HCV monoinfection, sex, age at liver biopsy, age at infection, duration of infection, ALT activity, and AST activity. The Hosmer-Lemeshow goodness-of-fit test was used to assess the model fit. Considering a strong correlation between ALT and AST, two separate models were constructed to avoid multicollinearity: model 1 including ALT and model 2 including AST.

A two-sided *p* value of <0.05 was considered significant. All statistical analyses were performed using MedCalc Statistical Software ver. 12.1.4.0 (MedCalc, Mariakerke, Belgium).

## Results

### Baseline characteristics of the study population

**Fig. 1:**
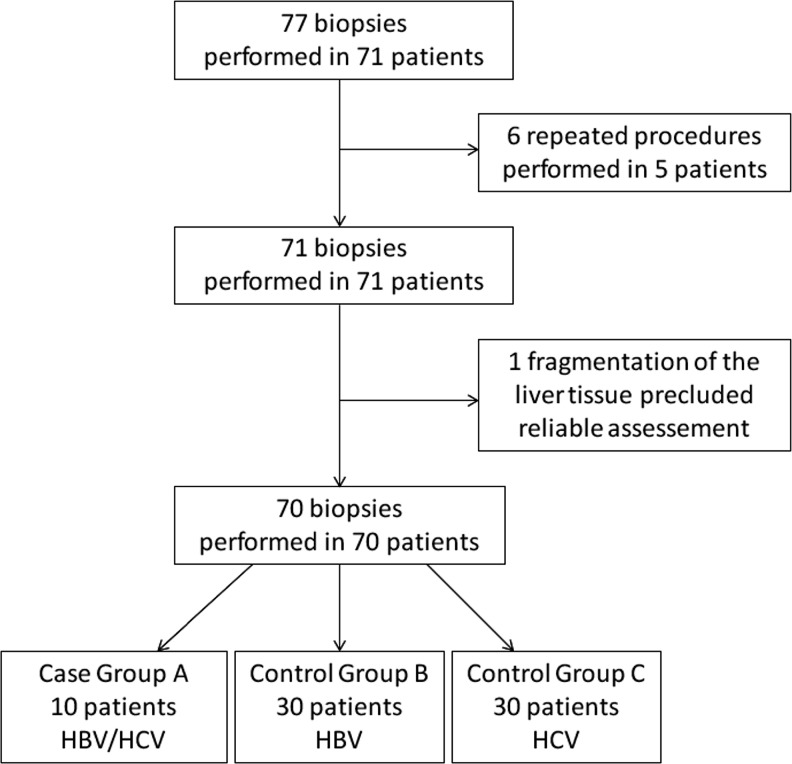
Flowchart of patient selection

A total of 77 liver biopsy specimens obtained from 71 consecutive children with chronic viral hepatitis were analyzed. Five children underwent a repeat liver biopsy, including one who had three biopsies. In these cases, only the initial biopsy was analyzed. In one case, fragmentation of the tissue precluded a reliable assessment of the specimen (Fig. 1). The final analysis comprised 70 treatment-naïve patients (48 boys and 22 girls, aged 5–17 years, mean 12.2 ± 3.1 years) divided into three groups: 10 (14 % of the study group) with chronic HBV/HCV coinfection, 30 (43 %) with chronic hepatitis B, and 30 (43 %) with chronic hepatitis C. Children with HBV/HCV coinfection (case group A) were compared to those with HBV monoinfection (control group B) and HCV monoinfection (control group C). The statistical analysis did not reveal any significant differences between case group A and the control groups with respect to the proportion of male/female patients, age at liver biopsy, duration of infection, age at infection, BMI, BMI *z*-score, HBV and HCV viral load, or mode of transmission (Table 1). Most subjects acquired the infection nosocomially. Ten percent of children in both control groups were vertically infected, but no case of vertical transmission was reported among patients with coinfection. The distribution of genotypes in children with HBV infection was as follows: A (50 %) and D (50 %). Genotype 1 (1a and 1b) was detected in 86 % of all children with HCV infection, genotype 3a in 9 %, and genotype 4 in 5 %.

**Table 1:** Demographic and biochemical characteristics of children enrolled in the study

Characteristics	Total	Case group A HBV/HCV	Control group B HBV	Control group C HCV	*p*		
					HBV/HCV vs. HBV	HBV/HCV vs. HCV	HBV vs. HCV
Number of patients	70	10	30	30			
Sex							
Male (%)	48 (69)	6 (60)	19 (63)	23 (77)	1.0	0.42	0.40
Female (%)	22 (31)	4 (40)	11 (37)	7 (23)			
Age at liver biopsy (years) (mean ± SD)	12.2 ± 3.1	12.6 ± 2.7	12.9 ± 2.5	11.5 ± 3.6	0.75	0.40	0.09
Duration of infection (years) (mean ± SD)	10.9 ± 3.4^a^	10.9 ± 3.6^b^	11.7 ± 2.5^c^	10.1 ± 4.0^d^	0.48	0.60	0.08
Age at infection^e^ (median (IQR))	0.1 (0.1–1.0)^a^	0.1 (0.1–0.1)^b^	0.1 (0.1–3.0)^c^	0.1 (0.1–0.1)^d^	0.79	0.79	0.60
Mode of transmission							
Vertical (%)	6 (9)	0	3 (10)	3 (10)	0.35	0.28	*0.003*
Nosocomial (surgery, hospitalization) (%)	38 (54)	6 (60)	22 (73)	10 (33)			
Blood transfusion (%)	22 (31)	3 (30)	3 (10)	16 (54)			
Unknown (%)	4 (6)	1 (10)	2 (7)	1 (3)			
ALT (median (IQR))	73 (47.3–113)	88.5 (45–126)	64 (51–103)	80 (36–122)	0.43	0.69	0.62
AST (median (IQR))	51 (36–72.3)	59 (38–68)	50 (38–65)	47 (36–80.8)	0.64	0.91	0.49
HBeAg							
Positive (%)	18/40 (45)	2 (20)	16 (53)	–	0.08	–	–
Negative (%)	22/40 (55)	8 (80)	14 (47)	–			
HBV DNA viral load (IU/ml) (median (IQR))	6.57 × 10^7^ (2.58 × 10^4^–1.72 × 10^8^)	1.7 × 10^4^ (4.99 × 10^2^–3.3 × 10^4^)	9.4 × 10^7^ (2.6 × 10^6^–2.45 × 10^8^)	–	0.17	–	–
HCV RNA viral load (IU/ml) (median (IQR))	7.22 × 10^5^ (3.80 × 10^5^–1.67 × 10^6^)	6.85 × 10^5^ (5.14 × 10^5^–1.5 × 10^6^)	–	8.12 × 10^5^ (3.4 × 10^5^–1.64 × 10^6^)	–	0.95	–
BMI (kg/m^2^) (mean ± SD)	19.6 ± 4.0	18.5 ± 2.6	19.3 ± 3.7	20.3 ± 4.6	0.52	0.23	0.33
BMI z-score (SD)							
Mean ± SD	0.29 ± 1.4	0.29 ± 1.5	0.1 ± 1.3	0.7 ± 1.5	0.47	0.07	0.08
>2 SD (obesity) (%)	10 (14)	0	3 (10)	7 (23)	0.55	0.16	0.17

^a^Data available for 66 patients

^b^Data available for nine patients

^c^Data available for 28 patients

^d^Data available for 29 patients

^e^Age of 0.1 year indicates horizontal infection during the neonatal period

### Liver histopathology

**Fig. 2:**
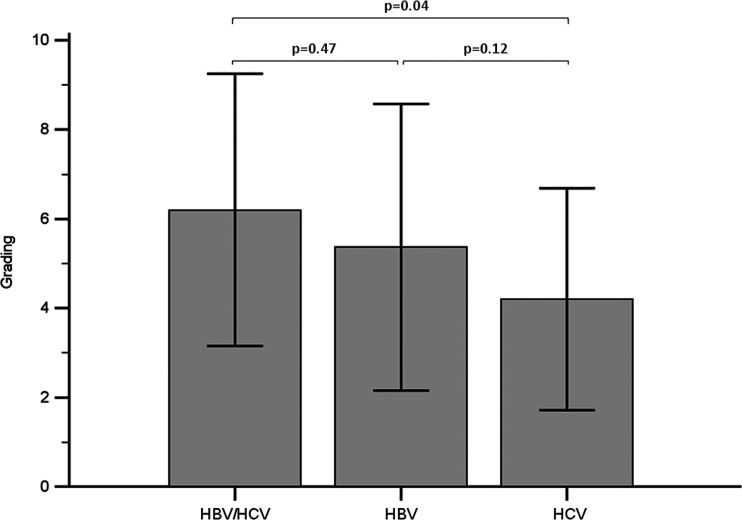
Mean grading in case group HBV/HCV compared to monoinfection control groups. *Error bars* indicate standard deviations

Children with HBV/HCV coinfection most often presented with mild to moderate necroinflammation and stage 2 fibrosis (Table 2). The mean grade was significantly higher in case group A compared to control group C (*p* = 0.04), but no difference was found with control group B (*p* = 0.47, Table 2, Fig. 2). In addition, a higher proportion of patients in case group A had moderate to severe necroinflammation compared to control group C (*p* = 0.05, Table 2), but not when compared to control group B (*p* = 0.2, Table 2). The mean staging values and proportions of patients with each staging score did not differ between the studied groups. The presence of lymphoid aggregates was confirmed in 25/70 (36 %) patients, and steatosis was found in 11/70 (16 %) patients in the study group. No significant differences were found in the prevalence of lymphadenoplasia and steatosis between case group A and both controls.

**Table 2:** Histopathological features of studied liver biopsies

Histopathological feature	Total (*n* = 70)	Case group A HBV/HCV (*n* = 10)	Control group B HBV (*N* = 30)	Control group C HCV (*N* = 30)	*p*		
					HBV/HCV vs. HBV	HBV/HCV vs. HCV	HBV vs. HCV
Grading of necroinflammation							
Mean ± SD	5.0 ± 2.9	6.2 ± 3.0	5.4 ± 3.4	4.2 ± 2.5	0.47	*0.04*	0.12
Minimal (0–3) (%)	18 (26)	2 (20)	7 (24)	9 (30)	0.2	*0.05*	0.22
Mild (4–8) (%)	43 (61)	5 (50)	18 (60)	20 (67)			
Moderate (9–12) (%)	8 (11)	3 (30)	4 (13)	1 (3)			
Severe (13–18) (%)	1 (3)	0	1 (3)	0			
Staging of fibrosis							
Mean ± SD	1.5 ± 0.9	1.7 ± 0.8	1.7 ± 0.9	1.2 ± 0.9	0.92	0.14	*0.036*
0 (%)	9 (13)	1 (10)	2 (7)	6 (20)	0.69	0.33	0.32
1 (%)	26 (37)	2 (20)	11 (36.5)	13 (43)			
2 (%)	26 (37)	6 (60)	11 (36.5)	9 (30)			
3 (%)	8 (11.5)	1 (10)	5 (17)	2 (7)			
4 (cirrhosis) (%)	1 (1.5)	0	1 (3)	0			
Grading (0–3) + staging (0) (%)	7 (10)	1 (10)	2 (7)	4 (13)	1.0	1.0	0.67
Grading (9–18) + staging (3–4) (%)	3 (4)	1 (10)	1 (3)	1 (3)	0.44	0.44	1.0

In a univariate analysis, both ALT and AST activities were associated with moderate to severe necroinflammation (grade ≥9 points, *p* = 0.0042 and *p* = 0.009, respectively, Table 3). Multivariate analysis revealed that HBV/HCV coinfection (*p* = 0.03 and *p* = 0.04 depending on model) and ALT (*p* = 0.003) and AST (*p* = 0.007) activity were independently associated with moderate to severe necroinflammatory activity (grade ≥9 points, Table 3). None of the analyzed parameters were predictors of severe fibrosis (staging ≥3 points).

**Table 3:** Factors associated with moderate to severe necroinflammatory activity (grading ≥9)

Parameter	Univariate analysis	Multivariate analysis model 1	Multivariate analysis model 2
	Odds ratio (95 % CI)	Odds ratio (95 % CI)	Odds ratio (95 % CI)
HBV monoinfection	7.0 (0.82–59.5), *p* = 0.07	–	–
HCV monoinfection	0.57 (0.14–2.3), *p* = 0.43	–	–
HBV/HCV coinfection	3.78 (0.77–18.6), *p* = 0.10	8.4 (1.2–57.5), *p* = 0.03	7.3 (1.09–48.6), *p* = 0.04
Age at infection	0.77 (0.44–1.34), *p* = 0.36	–	–
Duration of infection	1.27 (0.98–1.64), *p* = 0.07	–	–
Age at liver biopsy	1.23 (0.93–1.62), *p* = 0.14	–	–
ALT	1.02 (1.005–1.03), *p* = 0.0042	1.02 (1.007–1.03), *p* = 0.003	–
AST	1.03 (1.007–1.05), *p* = 0.009	–	1.03 (1.009–1.06), *p* = 0.007
Sex^a^	0.86 (0.19–3.81), *p* = 0.84	–	–
Model performance			
AUC		0.90 (0.80–0.96)	0.862 (0.758–0.933)
Hosmer-Lemeshow goodness-of-fit test^b^		10.7, 8, *p* = 0.22	12.9, 7, *p* = 0.07

Considering a strong correlation between ALT and AST, two separate models were constructed to avoid multicollinearity: model 1 including ALT and model 2 including AST

^a^For male sex

^b^Data are *χ* ^2^ statistic, degrees of freedom, and *p* value; a high *p* value indicates a good fit of the data with the model

**Fig. 3:**
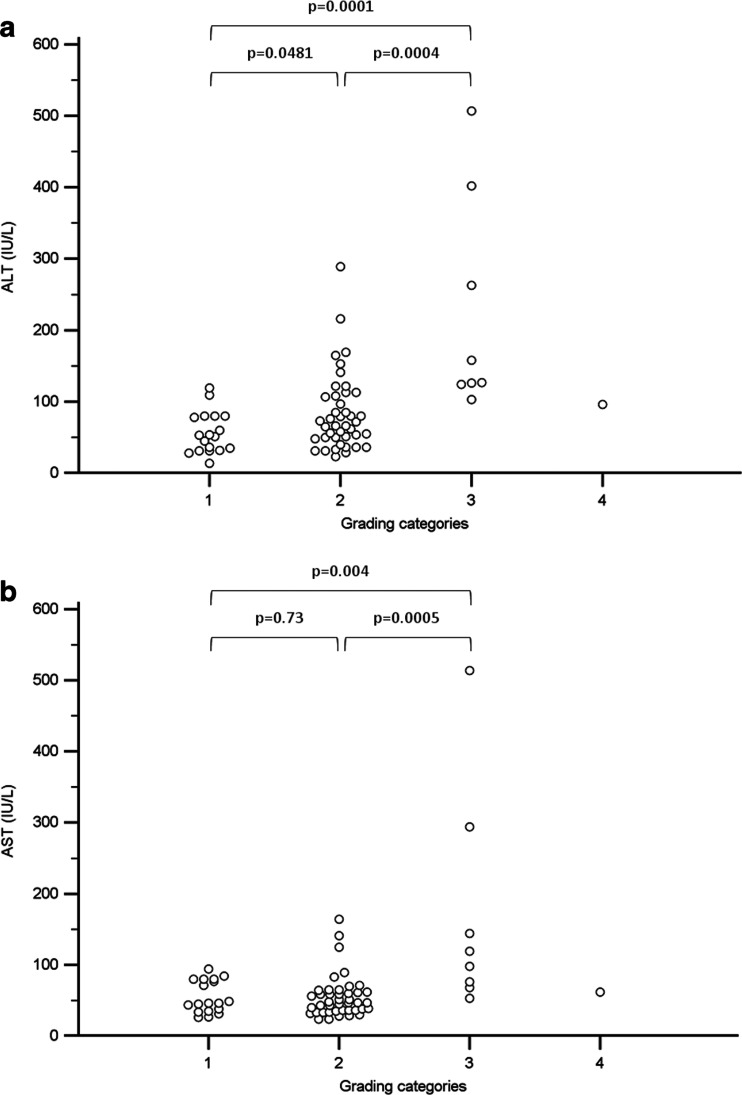
Aminotransferase levels based on necroinflammation grades: *1* (minimal, 0–3 points), *2* (mild, 4–8), *3* (moderate, 9–12), and *4* (severe, 13–18). **a** ALT versus grading. Kruskal-Wallis test *p* = 0.0003. **b** AST versus grading. Kruskal-Wallis test *p* = 0.005

We observed a trend toward more advanced fibrosis in patients with older age at liver biopsy (*p* = 0.06). However, we observed no differences in the age at infection (*p* = 0.43), duration of infection (*p* = 0.24), ALT activity (*p* = 0.18), and AST activity (*p* = 0.27) in subgroups divided according to fibrosis staging. On the other hand, ALT and AST activities were significantly different between subgroups with consecutive necroinflammation grades (Fig. 3). Age at liver biopsy, age at infection, and duration of infection did not differ between patients with different grades (*p* = 0.53, *p* = 0.83, and *p* = 0.35, respectively).

Histopathological evaluation did not reveal any significant changes in one patient with coinfection, which was similar to two children with HBV and four with HCV alone. In one patient with coinfection (10 %), both necroinflammation and fibrosis were advanced (grading 11, staging 3), compared to one (3 %) in control group B and one (3 %) in control group C. Cirrhosis was present in only one patient (a 14-year-old boy infected nosocomially with HBV at the age of 3 years, with ALT and AST serum levels between 1 and 1.5 ULN: ALT 54 IU/l and AST 44 IU/l).

## Discussion

Chronic viral hepatitis in childhood is usually a benign disease and does not lead to severe liver damage [10, 11]. However, in this study, we demonstrated a wide spectrum of histopathological lesions in children with chronic viral hepatitis, including mild to severe necroinflammation and fibrosis. Previous studies have shown that HBV/HCV coinfection in adults is associated with more severe liver disease than monoinfection with either virus [9, 23, 24]. However, data in children with coinfection are limited [22, 26, 27]. Morphological changes in patients with HBV/HCV coinfection in our study comprised lesions, which are common to the etiologies of both HBV and HCV [12].

In the present study, multivariate analysis revealed that HBV/HCV coinfection is an independent factor associated with a higher risk of moderate to severe necroinflammatory activity in children, which is a unique finding. No such correlation was found for severe fibrosis. In addition, we showed that necroinflammatory activity is more pronounced in children with HBV/HCV coinfection than in children with HCV monoinfection, but no difference was found when comparing coinfection to patients infected with HBV only. HBV and HCV are two different viruses that contain disparate genomes and distinct replication mechanisms. However, several different outcomes can occur when the liver is affected simultaneously by these two viruses, including synergistic damage and inhibited replication of the other virus [13]. Sagnelli et al. observed that HBV infection speeds up the progression of hepatitis related to HCV, despite the reciprocal inhibition of viral genomes [24]. However, a stimulating role of HCV in the progression of chronic hepatitis B is also possible [13, 17, 24, 28]. Similarly, in patients with HBV/HCV coinfection, the presence of HCV may improve clinical outcomes compared to the expected outcome of chronic hepatitis B alone [13].

The proportion of children in whom no significant morphological changes were found in the liver was relatively low (10 %) in the coinfection group and similar to the groups with monoinfection (7 % in control group B and 13 % in control group C). In contrast, Goodman et al. reported nearly normal biopsies or only minimal inflammation in 52 (43 %) out of 110 studied liver biopsies in children with chronic hepatitis C [10]. In our study, although the absolute percentages suggested a higher proportion of children with both advanced necroinflammation (grade ≥9 points) and fibrosis (staging ≥3 points) among the coinfected patients (10 %) compared to both control groups (3 % in both groups), the observed difference did not reach significance, probably due to the small numbers of patients in each group.

The presence of cirrhosis in only one patient precluded any reliable analysis concerning factors associated with cirrhosis in pediatric patients with chronic viral hepatitis. Nevertheless, the fact that we observed cirrhosis in a 14-year-old boy with an 11-year history of HBV infection is particularly important. Further studies are needed to assess the true incidence of cirrhosis in children with chronic viral hepatitis.

The mean HAI in our pediatric cohort with HBV/HCV coinfection was similar to that observed by Sagnelli et al. in 27 adult patients with coinfection (6.2 ± 3.0 vs. 5.8 ± 3.4 points, *p* = 0.75). The same was found for fibrosis (mean staging score 1.7 ± 0.8 in our study vs. 2.1 ± 1.2 in adults, *p* = 0.25) or control groups B and C [24]. These facts underscore that HBV/HCV coinfection in children is as aggressive as in adults. However, further prospective studies are needed to directly compare histopathological changes in children and adults coinfected with HBV/HCV.

Conflicting data exist on the influence of patient age or duration of infection with both HBV and HCV on histopathological lesions in the liver [1, 6, 10, 20, 21]. In the present study, we found a trend toward higher staging in patients with older age at liver biopsy (*p* = 0.06). However, we did not confirm the correlation between age at infection or duration of infection and the severity of histopathological changes in the liver. Thus, severe liver damage as a consequence of chronic hepatitis is possible even in young children, irrespective of the duration of infection or age at infection. These facts call into question the benign nature of chronic hepatitis in children.

Data on the relationship between aminotransferase activity and the severity of histopathological features in the liver are also conflicting. According to Hudacko and Theise, serum transaminase levels are increased in almost all patients with chronic hepatitis, but the levels do not necessarily reflect the severity of necroinflammatory activity [14]. Up to 30 % of HBV-infected patients with persistently normal ALT levels are estimated to have significant fibrosis, and 10 % may have bridging fibrosis or cirrhosis, which indicates that normal ALT does not exclude progressive liver disease [10, 18]. We demonstrated that ALT and AST activities are independently associated with moderate to severe necroinflammation (grade ≥9 points), but not fibrosis. Similar observations have been made by other authors in patients with chronic hepatitis C [1, 10, 19]. Interestingly, Badizadegan et al. showed a significant correlation between ALT levels and HAI in children with chronic hepatitis C, but the strength of the correlation depended on the grading scheme used for histopathological assessment [1].

### Limitations

As a cross-sectional study, our study did not permit us to distinguish between causes and effects. Thus, the causal relationship between variables demonstrating an association in our study needs to be confirmed in prospective cohort studies. In addition, the slides were read by one pathologist. Taking into consideration an interobserver variation, an additional pathologist could make the results more reliable. A limitation of the study was a relatively small number of subjects in the case group. In particular, the conclusion that HBV/HCV coinfection was associated with higher necroinflammatory activity when compared to HCV monoinfection, whereas no difference existed with HBV monoinfection, may result from the low number of children with coinfection taken into consideration. However, papers on children are lacking in this field, and the information regarding the results of liver biopsies in children with coinfection should be considered unique, whereas comparisons between the case and control groups may be undermined by the aforesaid limitations.

In conclusion, children with HBV/HCV coinfection presented with a wide range of histopathological features in the liver, including severe necroinflammation and fibrosis, irrespective of age at liver biopsy, duration, and age at infection. HBV/HCV coinfection was associated independently with moderate to severe necroinflammation and led to significantly higher necroinflammatory activity compared to HCV, but no difference was found with HBV monoinfection. However, HBV/HCV coinfection did not enhance fibrosis staging compared to monoinfection. Moreover, high aminotransferase levels were good predictors of the degree of necroinflammation, but not fibrosis.

## Abbreviations

ALT: Alanine aminotransferase
AST: Aspartate aminotransferase
BMI: Body mass index
HAI: Histologic activity index
HBV: Hepatitis B virus
HCV: Hepatitis C virus
IQR: Interquartile range
RT-PCR: Real-time polymerase chain reaction
SD: Standard deviation
ULN: Upper limit of normal
